# Our Secretaries of Agriculture

**Published:** 1897-03

**Authors:** 


### EDITORIAL.

### OUR SECRETARIES OF AGRICULTURE.

THE present month will witness the closing acts of the present national administration of the Department of Agriculture and the incoming of the new. Much has been said, more has been written, of the present administration than any of its predecessors for many years. The greatest diversity of opinion has existed as to the merits of the work accomplished and its methods of performance. But now that it has been completed we can look upon it with less personal feeling and deal with it as a whole. First, it must be looked at from the standpoint of a Democratic administration of public affairs, and in two cardinal points—that of economy and a full equivalent for the money expended and in a just return of labor for salaries paid—it stands out single and alone as one of the best managed and directed the country has ever witnessed. Saving in gross amount thousands upon thousands of dollars that are wrung from the people by indirect taxation, it has at the same time secured the most efficient services from its employés of any administration in twenty years. As the work has increased in volume, the responsibilities have become greater and more exacting and the conditions more severe and restrictive, in a great measure the result of foreign proscriptive legislation aimed at our export food-products. Still the cost of doing this work has been lessened, and it has been performed with increased efficiency and has enlarged in almost every direction the foreign markets for our surplus food-products. This is wholly democratic, and there can be no difference of opinion among all good citizens as to its wisdom and worth at this time, and of the good lessons taught thereby by our parental government. Every aspect of paternalism has been vigorously opposed; and this, too, is democratic, thereby encouraging and fostering among our people a feeling of self-reliance and the largest liberty in the principle of self-government. The cost of inspection of foods for foreign shipment has been strenuously advised to be placed upon those who profit by the foreign markets and the infamous seed-scandal been laid open to public light, which will in the near future so solidify public opinion against its perpetuation that it will be wiped off our statute books as a blot and scandal upon the fair name of our national government. While there is and has been a persistent disposition for Republican rule and power to claim all the honor and any benefits accruing from the establishment of a true civil service, it was left in this department to a Democratic official to enforce the spirit and intent of true civil service throughout the Department of Agriculture. Without fear or favor Secretary Morton has earnestly, conscientiously, and with steadfast devotion to duty as an executive officer overcome every obstacle and condition until he has established civil service, or the merit system, in every branch of the department, from the highest to the lowest official, and thus secured to the government the best service and the highest efficiency it has received for a score of years. He has made these offices secure from removal on political grounds, and he has not demanded political services for their retention. Adherence to party or services to State or local political bosses add no strength to a desired entry to public service; and while this may not be democratic teaching, may controvert democratic doctrine as taught by the founders of the Democratic party, let it not be gainsaid that all the more honor to his intergity for the enforcement of so wise a measure, which has brought so much good to all. In his final report to the President he has gone further in the advocacy of the system by calling attention to the inadequate payment of special services of a scientific character rendered the government and to the injustice done these employés when compared with the more than well-paid clerical force, whose positions and salaries have ever been sought for and considered as sinecures. Much more might be well said of the thorough and wise administration of this department in the interest of true reform and advancement for the veterinary profession; but sufficient has been alluded to, and a grateful appreciation of the changes in this department will long follow our outgoing Secretary as a contributor in no small measure to the welfare of our calling. While speeding the parting guest with the sincere thanks of a determined and aggressive profession, winning its way by sincere and devoted work, aided by such well-directed assistance as we have enjoyed at Secretary Morton's hands, we welcome the incoming Secretary, Prof. James Wilson, with the full appreciation of the grave responsibilities and trials before him, and when the evening of his administration shall arrive we trust we may be able to speak with as much praise and with the same feeling of appreciation for an advancement on his part of the good work of his predecessor.

---

THE to-be Secretary of Agriculture, Prof. James Wilson, of Ames, Iowa, Professor of Agriculture at Ames, proves to be an appointment generally acceptable to all interests involved. Thoroughly versed in agriculture from boyhood up, his value as an efficient instructor was recognized by the State Agricultural College in his election to the Chair of Agriculture, which he has efficiently filled and greatly promoted the growth and advancement of that institution. Friendly to and interested in its Veterinary Department, the profession is assured of a friend in this department of our government, and one who has kept himself familiar with the advancement of our profession and fully cognizant of its worth and usefulness. We shall look forward to the steady enlargement of this department of the Bureau of Agriculture, that the live-stock industry of the country may be advanced and increased in worth to our people in every wise and guarded direction.

